# Impact of a Virtual Reality-Based Simulation on Empathy and Attitudes Toward Schizophrenia

**DOI:** 10.3389/fpsyg.2022.814984

**Published:** 2022-05-04

**Authors:** Antonio J. Marques, Paulo Gomes Veloso, Margarida Araújo, Raquel Simões de Almeida, António Correia, Javier Pereira, Cristina Queiros, Rui Pimenta, Anabela S. Pereira, Carlos F. Silva

**Affiliations:** ^1^Center for Rehabilitation Research, School of Health, Polytechnic of Porto, Porto, Portugal; ^2^Santa Maria Health School, Porto, Portugal; ^3^CITIC Research Center, University of A Coruña, A Coruña, Spain; ^4^Faculty of Psychology and Education Science, University of Porto, Porto, Portugal; ^5^School of Health, Polytechnic of Porto, Portugal and CEISUC, University of Coimbra, Porto, Portugal; ^6^Department of Education and Psychology, University of Aveiro, Aveiro, Portugal

**Keywords:** empathy, attitudes, schizophrenia, virtual reality, stigma

## Abstract

Virtual Reality (VR) has been identified as one of the most promising resources for developing empathy towards stigmatized groups as it allows individuals to experience a situation close to reality from another person’s perspective. This quasi-experimental study aimed to examine the impact on empathy, knowledge, and attitudes towards people with schizophrenia of a VR simulation that reproduces the experience of psychotic symptoms while performing a cognitive task compared with watching a 2D video and, thus, how these experiences could reduce stigma towards people diagnosed with schizophrenia. The sample comprised of 102 higher education health students, distributed by the experimental and control groups. The impact of the program was measured by completing multiple questionnaires on levels of empathy, attitudes, and mental health knowledge. Both methods (VR and 2D video) were, to a certain extent, effective. However, VR was more effective at eliciting attitudes and knowledge change compared to the control group. These findings suggest that not only VR but also 2D videos could be interesting strategies to enhance empathy and improve attitudes towards people with schizophrenia in higher education health students.

## Introduction

Epidemiological studies consistently demonstrate, over time, the higher prevalence of neuropsychiatric disorders worldwide and their impact on the functionality, social participation, and quality of life of the people who experience them (Carvalho et al., 2016; Carvalho, 2017; World Health Organization Regional Office for Europe, 2018). Schizophrenia, currently considered as one of the most serious neuropsychiatric disorders, is associated with severe deficits in cognitive, social, and occupational functioning, compromising the individual’s abilities in their daily life (American Psychiatric Association, 2013; Kahn et al., 2015; Owen et al., 2016; Birur et al., 2017).

Despite the increasing trend in the prevalence of mental illness, people diagnosed with schizophrenia continue to face enormous difficulties and barriers to social participation. They are often victims of the most varied forms of prejudice, stigma, discrimination, and exclusion by society (Thornicroft et al., 2009; Corrigan, 2016; Patel et al., 2018). Stigma leads to a reduction in equal opportunities, an obstacle to adequate social integration and a negative influence on the possibility of recovery (Kleim et al., 2008; Corrigan, 2016; Clay et al., 2020). In addition, it also stems from the fear of the unknown and negative beliefs rooted in society, thus mirroring the lack of knowledge, and understanding about mental disorders, giving rise to attitudes based on prejudice and promoting discrimination (Corrigan and Shapiro, 2010; Corrigan, 2016). The stigmatizing images generated around these people also lead to high levels of social distance (Kutcher et al., 2016; Degan et al., 2019; Clay et al., 2020).

Stigma involves three elements; a lack of knowledge (ignorance), negative attitudes (prejudice), and people behaving in ways that disadvantage the stigmatized person (discrimination). Two main types of stigma occur with mental health problems, social stigma and self-stigma. Social stigma, also called public stigma, refers to negative stereotypes of those with a mental health problem. These stereotypes come to define the person, mark them out as different, and prevent them being seen as an individual. Self-stigma occurs when a person internalizes negative stereotypes. This can cause low self-esteem, shame, and hopelessness. Both types of stigma can lead a person to avoid seeking help for their mental health problem due to embarrassment or fear of being shunned or rejected (Rezayat et al., 2019). Stigma is present in the lives of people diagnosed with schizophrenia, being considered as a “second illness,” since 40% of this population feel strongly stigmatized (Valery and Prouteau, 2020). According to Corrigan (2016), contact with the socially fragile or excluded person is the most effective strategy to reduce stigma since that reveals significant changes in attitudes and behavioral intentions. As a result, the population might develop an empathetic perspective.

In its cognitive and affective dimensions, empathy involves the ability of an individual to relate to and put themselves in someone else’s place, recognizing, comprehending, and acknowledging someone’s conduct and background. Thus, they assumed an understanding of their point of view, their expressions and how they respond to various situations, and the very experience of their emotions at a personal level (Christofi and Michael-Grigoriou, 2017; Bertrand et al., 2018; Queirós et al., 2018; Tong et al., 2020). This capacity is vital to have success in social interactions, since it allows to understand someone else’s emotions better (Herrera et al., 2018).

Despite empathy being a topic that has been gaining more attention, it has been shown that the teaching of empathy in colleges and universities has been decreasing; in fact, there is some controversial evidence that showed that during medical training, the empathy levels of the students have been diminishing (Igde and Sahin, 2017; Triffaux et al., 2019). Even though that there are different positions about the levels of empathy during future health professionals training, it is a consensus that there is a need to teach empathy to health students, since it is believed that would help them improve their communication skills and develop theirs attitudes toward patients (Sulzer et al., 2016; Tong et al., 2020).

Several studies point to perspective-taking exercises as one of the best ways to foster empathy. This approach enables people to understand the other person’s internal states by cognitively placing themselves in their perspective (Decety et al., 2012; Decety and Cowell, 2015; Christofi and Michael-Grigoriou, 2017; Bertrand et al., 2018; Loon et al., 2018). The basis of this method is to allow the participant to use their imagination to try to understand someone’s perspective. Although there are some limits, as it can be an exhausting technique that requires a lot of cognitive effort, which may lead the participant to avoid performing this activity (Bailenson, 2018). In addition, the individual imagination can be limited if the participant has reduced contact and/or wrong information about the target population (Herrera et al., 2018; Rueda and Lara, 2020).

Given its importance and its positive effects on social relationships in promoting the wellbeing of others, the development of altruistic behavior, perspective-taking, and prosocial behaviors, researchers have been trying to find new ways to increase empathy (Decety, 2010; Dunfield, 2014; Decety and Cowell, 2015; Decety et al., 2016). There are several ways to apply the method of perspective-taking, such as role-playing, mental simulations, narrative constructions, and videogames, among others (Rueda and Lara, 2020). The videogames send sensory stimuli, such as visual, auditory, and sometimes haptic, requiring the user to recruit theirs visual, auditory, and proprioceptive systems. Adding these characteristics to the virtual narrative, virtual video games, specifically using Virtual Reality (VR), have been considered an effective way to promote empathy (Herrera et al., 2018). VR overcomes the limitations previously described of the traditional methods, since the users do not need to imagine the other person’s perspective; they only need to focus on the experience that they are going through (Rueda and Lara, 2020).

In this sense, VR has been used as a tool to promote empathy toward stigmatized groups since it allows individuals to experience a situation close to reality from another person’s perspective (Christofi and Michael-Grigoriou, 2017; Schutte and Stilinović, 2017; Bertrand et al., 2018; Formosa et al., 2020). It has been shown that videogames have the potential to promote empathy despite players being primarily interested in winning. Since VR games elicit the feeling of presence, the users’ subjective feeling of being inside an immersive environment allows them to understand perspectives other than their own more genuinely when compared to a 2D video; therefore, this technology shows a greater facility to increase empathy levels compared with traditional forms (Herrera et al., 2018; Tong et al., 2020). VR is a technology that creates a 3D environment on a computer where the user can interact with digital scenarios, objects, and avatars with the use of some gadgets such as headpieces and gloves. This technology has a lot of application in several areas, where the user can have virtual training, for example, for preparation for surgeries or dealing with stressful situations. In health education, VR has also been applied to virtual patients that are used to grow clinical thinking and to teach soft skills like communication skills or empathy (Freeman et al., 2017; Quail and Boyle, 2019; Haowen et al., 2021). Thus, this technology is a promising training tool in the health field since it allows individuals a possibility of immersion and presence in a virtual world, where they can interact and explore the environment in real-time (Aguinas et al., 2001; De la Peña et al., 2010; Izard et al., 2018). Therefore, VR provides the users with a strong sense of immersion, presence, and interaction with the environment, leading them to understand perspectives other than their own. This intervention may help perceive and interpret what the other person thinks, feels, and expresses in a specific day-to-day situation (Schutte and Stilinović, 2017). The higher interactivity has been associated with higher feelings of presence, which consequently can promote empathy more effectively. In this way, VR has been called the “ultimate empathy machine” because of its properties that elicit presence and therefore allow the user to encounter another point of view (Herrera et al., 2018). Perspective-taking is more effective than providing data to feel empathy. VR embodied perspective-taking can foster empathic abilities, allowing people not only to metaphorically walk in another person’s virtual shoes, but also to literally embody the virtual representation of the specific social target in whom they wish to increase empathy (Rueda and Lara, 2020). An increase in empathy is also possible due to reducing prejudice and social distance toward people diagnosed with schizophrenia (Slater and Sanchez-Vives, 2016; Christofi and Michael-Grigoriou, 2017; Tham et al., 2018).

Since schizophrenia affects over 20 million people in the world and stigma and discrimination toward this mental health problem are still frequent (World Health Organization, 2019), it is urgent to create anti-stigma interventions to help these patients to deal with their health condition (Gaebel et al., 2020). For a healthcare professional, it is vital to have empathy and compassion toward their patient regardless of their condition. Increasing these capacities during health school should be an essential aim for the institutions that could have or accentuate empathy in its vital objective (Louie et al., 2018). Also, it is believed that emotional regulation is linked to empathy, since high emotion regulation capacities have been related with mild levels of empathic concern. Changes in both heart rate and galvanic skin response are the responsibility of the autonomic nervous system; thus, some studies argue that emotion regulation may be associated with changes in situational empathy and autonomic response (Jauniaux et al., 2020; Sassenrath et al., 2021).

As VR is being characterized as an “empathy machine,” the present study aims to examine the impact on empathy, knowledge, and attitudes toward people with schizophrenia of a VR simulation that reproduces the experience of psychotic symptoms while performing a cognitive task compared with watching a 2D video and, thus, how these experiences could reduce stigma toward people diagnosed with schizophrenia. Thus, it was hypothesized that the VR experience (compared with the control condition) will as:

*H1*: Increases empathy toward people with schizophrenia.

*H2*: Increases positive attitudes toward people with schizophrenia.

*H3*: Increases mental health knowledge.

*H4*: Results in stronger physiological reaction than watching the same scenario as a 2D video.

## Materials and Methods

### Study Design

In this quasi-experimental study, two sample groups were defined, randomized, and evaluated two times, following a pre- and post-test methodology (Andrews and Likis, 2015).

### Participants

The sample was composed by 102 students from the School of Health—Polytechnic of Porto, Portugal (20 male and 82 female), divided half for the control group and half for the experimental group. Their ages varied from 18 to 47. Each group included 51 participants, 41 women and 10 men, with an average age ± SD of 21.75 ± 3.99 for the experimental group, while the control group had an average age of 20.63 ± 2.78. No difference was found between groups (*p* = 0,052). Although the sample does not claim to be representative of the population, it includes more women than men, as it reflects the reality of the student population at this organization (according to School of Health internal information management system, they have 2,869 students enrolled with the following distribution: 2,169 women and 678 men) and in other higher education health institutions in Portugal, which is made up of a large majority of women.

As the inclusion criteria for the study, participants had to be health students from the School of Health—Polytechnic of Porto and be 18 years old or older. In addition, exclusion criteria included having health issues that could compromise the virtual reality experience, such as epilepsy and labyrinthitis.

### Instruments and Technologies

#### Sociodemographic Questionnaire

The Sociodemographic Questionnaire was created by the research team to include questions about the participant. The questions include sex, age, nationality, and academic level, if they had a diagnosis of a mental health problem or if they knew someone who has been diagnosed with schizophrenia and if yes, how much time do they spend with that person.

#### Questionnaire of Cognitive and Affective Empathy

The Questionnaire of Cognitive and Affective Empathy (QCAE; Reniers et al., 2011; Queirós et al., 2018) is a self-reported measure of adult cognitive and affective empathy in mental illness. It consists of 31 items, with multiple-choice responses on a four-point Likert-type: “1 = Strongly Disagree” and “4 = Strongly Agree.” The dimension of affective empathy evaluates the ability to be sensitive and experience the emotional state of the other. In contrast, cognitive empathy evaluates the ability to understand the emotional state of the other. Affective empathy is subdivided into *Emotion Contagion*, *Proximal Responsivity*, and *Peripheral Responsivity*. *Emotion Contagion* is characterized by the ability to mirror other people’s emotional states automatically (e.g., “I am happy when I am with a cheerful group and sad when the others are glum”). *Proximal Responsivity* is the emotional state enacted through the perception of the sentiments and humors of a close relative (e.g., “Friends talk to me about their problems as they say that I am very understanding”). Finally, *Peripheral Responsivity* is the emotional state passed through the perception of a close relative’s feelings and senses of humor (e.g., “I often get deeply involved with the feelings of a character in a film, play, or novel”). Cognitive empathy is subdivided into *Perspective-Taking* and *Online Simulation*. The *Perspective-Taking* dimension is the ability to infer things from someone else’s point of view (e.g., “I am quick to spot when someone in a group is feeling awkward or uncomfortable”). In contrast, *Online Simulation* is the endeavor to put oneself in someone else’s shoes and infer your emotional state (e.g., “I find it easy to put myself in somebody else’s shoes”). On QCAE questionnaire, higher scores correspond to higher level of cognitive and affective empathy.

#### Empathic Feelings for People Suffering From Schizophrenia

The Empathic Feelings for People Suffering from Schizophrenia questionnaire (Kalyanaraman et al., 2010) was applied to assess how participants describe their emotions toward schizophrenia through 12 adjectives (e.g., “Sympathetic,” “Compassionate,” “Confused,” “Afraid,” and “Anxious”). These are rated on a seven-point scale, where 1 corresponds to “Not at all” and 7 corresponds to “Extremely.” On this scale, higher scores correspond to a more positive attitude.

#### Attitudes Toward People With Schizophrenia

The Attitudes Toward People with Schizophrenia questionnaire (Kalyanaraman et al., 2010) was applied to assess participants’ attitudes toward people diagnosed with schizophrenia. It consists of seven items (e.g., “How much personally do you care about the plight of people with schizophrenia?”) on a nine-point scale, where 1 corresponds to “Strongly disagree,” “Very,” “Extremely negative,” or “Not at all important” and 9 corresponds to “Strongly agree,” “Not at all,” “Extremely positive,” or “Very important,” where higher scores correspond to a more positive attitude.

#### The Mental Health Knowledge Schedule

The Mental Health Knowledge Schedule (MAKS; Camarneiro, 2018) is an instrument that allows the assessment of mental health knowledge. It consists of two parts, comprising 12 items, classified on a five-point Likert-type scale, where 1 corresponds to “strongly disagree” and 5 to “strongly agree,” where higher scores correspond to higher levels of knowledge. The first part comprises six items covering various areas of knowledge about factors associated with mental health: help-seeking, employment, recognition, support, treatment, and recovery, and is most closely related to stigma. The second part integrates the remaining six items and assesses knowledge about mental disorders. On this scale, higher scores correspond to a more knowledge about mental health.

#### Psychophysiological Data

To collect psychophysiological parameters, the commercial equipment Biopac Student Lab System MP36 was used. The Electrodermal Activity (EDA) and Heart Rate (HR) signals were analyzed using the AcqKnowledge software and *a posteriori* extracted and statistically analyzed (Andreassi, 2006; Braithwaite et al., 2013). These data were recorded in both groups (control group and experimental group). To monitor and collect psychophysiological parameters, two electrodes were placed on the participants: one on the index finger and one on the thumb of the palmar region of the left hand. These two electrodes measure the Electrodermal Activity (EDA) signal. In addition, three vinyl electrodes, one on the right shoulder, one on the left shoulder, and one on the sternum, measure the heart rate (ECG). The equipment used to monitor and collect these physiological parameters was the Biopac Student Lab System MP36. This equipment was connected to a computer with two channels: one channel for ECG and another for EDA.

### Procedures

The present study was conducted at Psychosocial Rehabilitation Laboratory (LabRP). All participants have previously completed an online pre-test questionnaire on the Google Forms platform, with the following sections: (1) sociodemographic data, which included questions regarding the contact participants may have with people diagnosed with mental health problems; (2) Questionnaire of Cognitive and Affective Empathy—QCAE; (3) Empathic Feelings for People Suffering from Schizophrenia; (4) Attitudes Toward People with Schizophrenia; and (5) The Mental Health Knowledge Schedule—MAKS.

Participants (Table 1) were randomly assigned to one of the two study groups, and the session was scheduled according to each participant’s availability.

**Figure 1:**
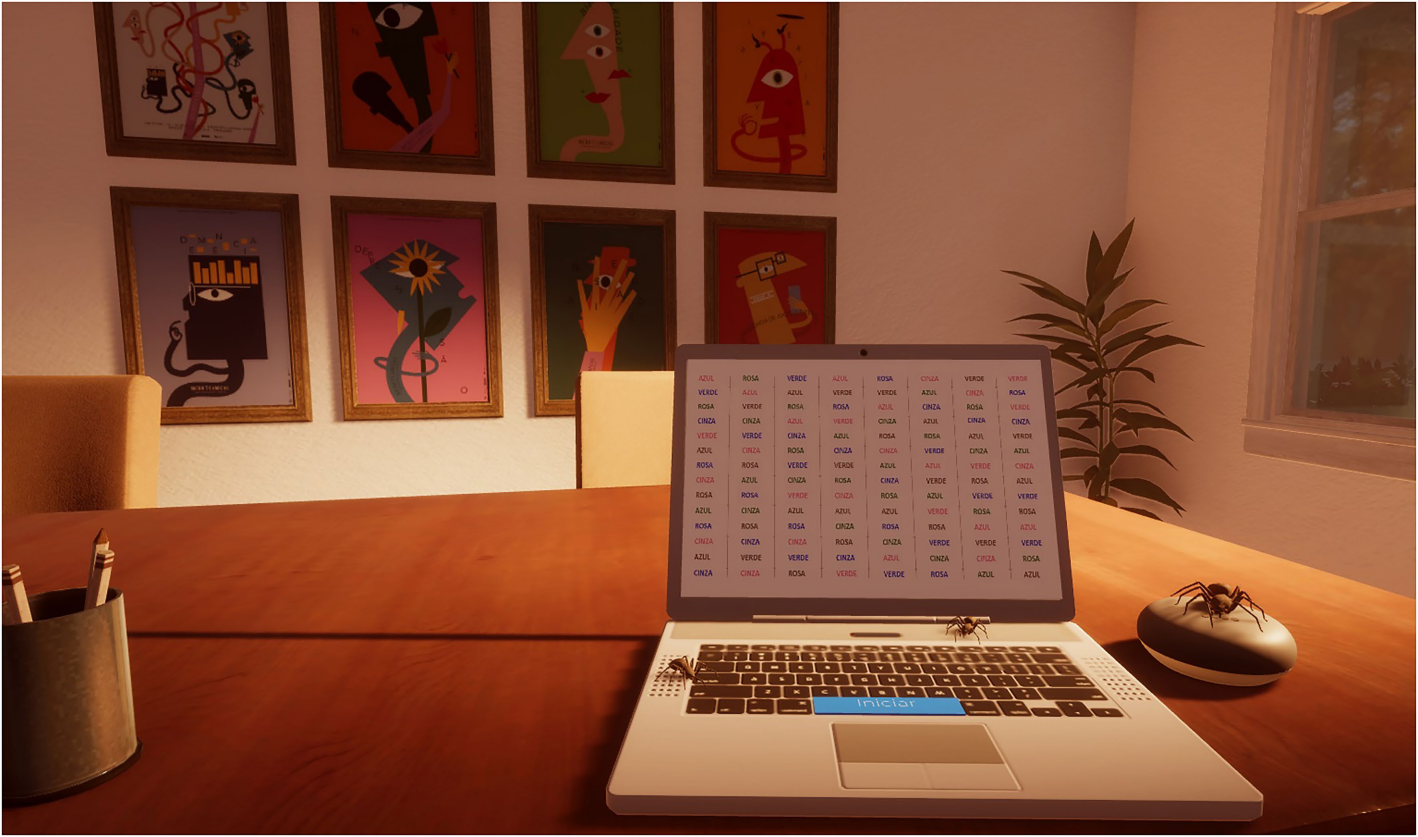
Simulation environment.

In the experimental group, participants were virtually exposed to typical positive symptoms of schizophrenia while performing a cognitive task [the authors choose the standard procedure of the Stroop Color and Word Test (Stroop, 1935)—it requires individuals to view a list of words that are presented in a different color than the meaning of the word. Participants are asked with naming verbally the color of the word, not the word itself]. The Stroop Test was chosen because is a one of the most used neuropsychological assessment tools, which allows inferences about attentional measures, specifically selective attention (Periáñez et al., 2021). The purpose was to accurately show the progressive difficulty in performing the test (a cognitive task) with the increasing delusions and hallucinations. The immersion and presence in the virtual environment were achieved using Oculus Quest 1. The VR environment was designed very similar to the room where the experiment was carried out to maximize the participant’s immersion and sense of presence (Figure 1). Participants represented a self-avatar, interacted with a virtual computer (in which they made the Stroop Test), and sat in a rotating chair, allowing them to observe the environment in all directions. The experiment began with four colored rectangles appearing in the virtual computer. Participants had to identify the colors of each rectangle. This stage had the purpose of guaranteeing the subject’s capacity of color recognition and color designation, as well as getting participants familiar with the virtual setting. After these onboarding phase started the virtual version of Stroop Color and Word Test. Participants were exposed progressively to external stimulus simulating schizophrenia symptoms while performing the Stroop Color and Word Test (e.g., voices in the background, hallucinations of moving frames, spiders, and visual effects, such as blur, tunnel vision, and sensitivity to glare).

**Table 1:** Sample sociodemographic characterization.

		Sample *n* = 102		Control group *n* = 51		Experimental group *n* = 51	
		Mean/*SD*	Min-Max	Mean/*SD*	Min-Max	Mean/*SD*	Min-Max
Age (years)		21.19 ± 3.47	18–47	20.63 ± 2.78	18–30	21.75 ± 3.99	18–47
		Frequency	%	Frequency	%	Frequency	%
Gender	Male	20	19.6%	10	19.6%	10	19.6%
	Female	82	80.4%	41	80.4%	41	80.4%
Academic degree	Graduate	102	100%	51	100%	51	100%

The protocol was the same in the control and in the experimental groups and the external stimulus (the room, the presence of auditory hallucinations and spiders, visual effects on the television, etc.) was similar with the difference that the control group viewed a 2D video and the experimental group was immersed in the virtual environment, i.e., for the control group, it was implemented the same narrative, however through a 2D video, without interaction and immersion. While the experimental group had the opportunity to experience virtually the positive symptoms of the schizophrenia and their impact on the cognitive performance, the control group did not have the same opportunity as they just saw a 2D video.

To collect psychophysiological parameters, the electrodes were connected to the user right before the start of the exposure. After the confirmation of the quality of the biosignals, the recording begins as the user puts the VR headset. The recording of biological signals occurs throughout all the exposure. The data were analyzed right after the exposure and at the end of all the experiments; the statistical analysis was carried out.

The exposure lasted 7 min in both groups. In the end, the participants of both groups answered an online post-test questionnaire with the following sections: (1) Questionnaire of Cognitive and Affective Empathy—QCAE; (2) Empathic Feelings for People Suffering from Schizophrenia; (3) Attitudes Toward People with Schizophrenia; and (4) The Mental Health Knowledge Schedule—MAKS.

### Statistical Analyses

All statistical tests were performed using the IBM SPSS Statistics 27 software. The sample’s sociodemographic characterization, descriptive statistics, absolute frequency, relative frequency, mean, and SD were calculated. The statistical analysis of the physiological data was carried out using an average, the maximum and the minimum value of each variable during the 7 min of exposure.

The significance of the improvements between the control group and the experimental group was assessed using a Student *t*-test for independent samples. The significance of the improvements between the pre-test (Moment 1) and post-test (Moment 2) time points was evaluated using a paired sample *t*-Student test. The assumptions of this method, specifically normality and homogeneity, were evaluated. The homogeneity of variances was verified using Levene’s test. Normality was assumed, following the Central Limit Theorem, since both groups have a good size dimension (*N* = 102; *n*_1_ = 51; and *n*_2_ = 51; Marôco, 2018).

## Results

**Figure 2:**
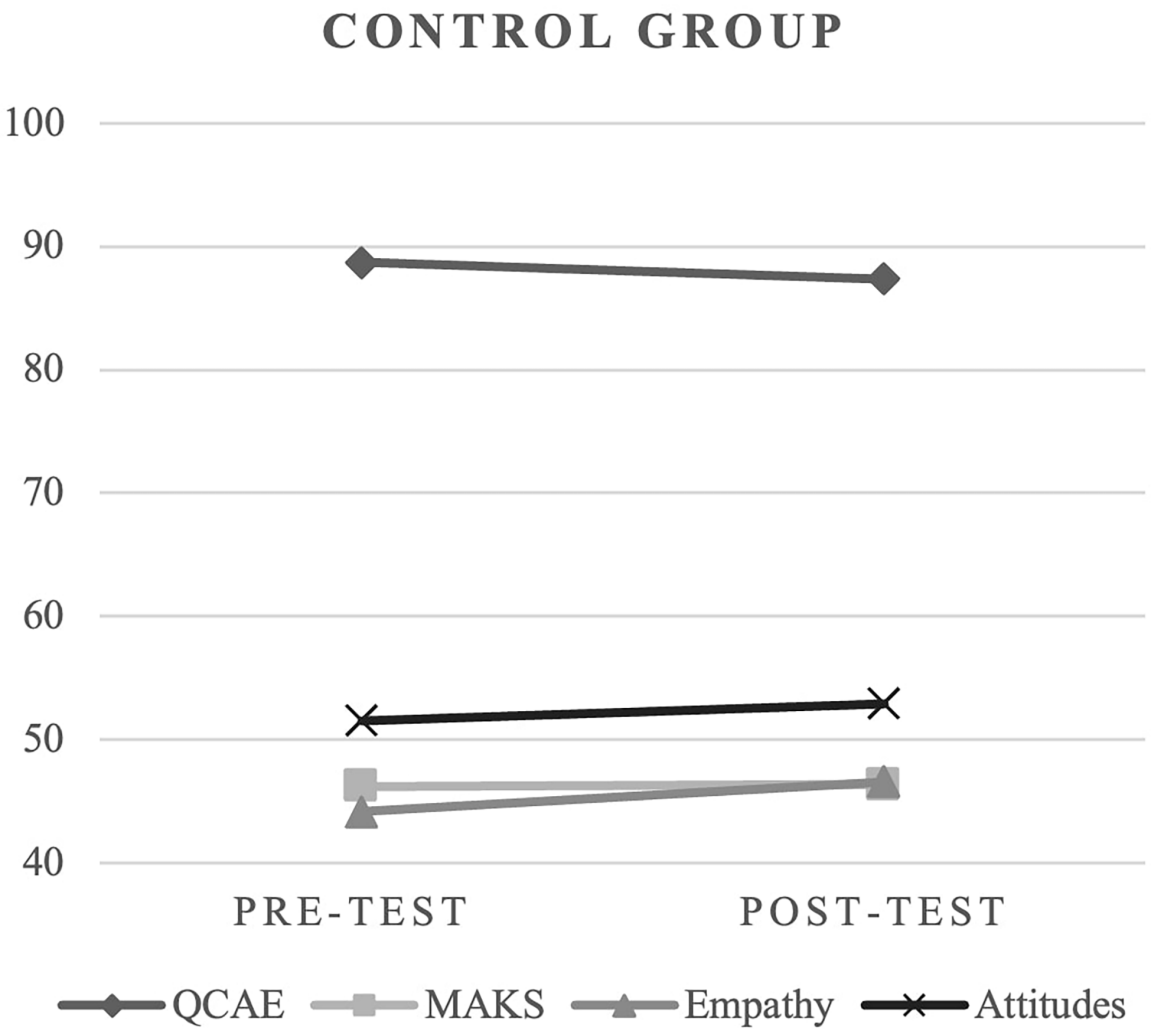
Comparative analysis in control group.

**Figure 3:**
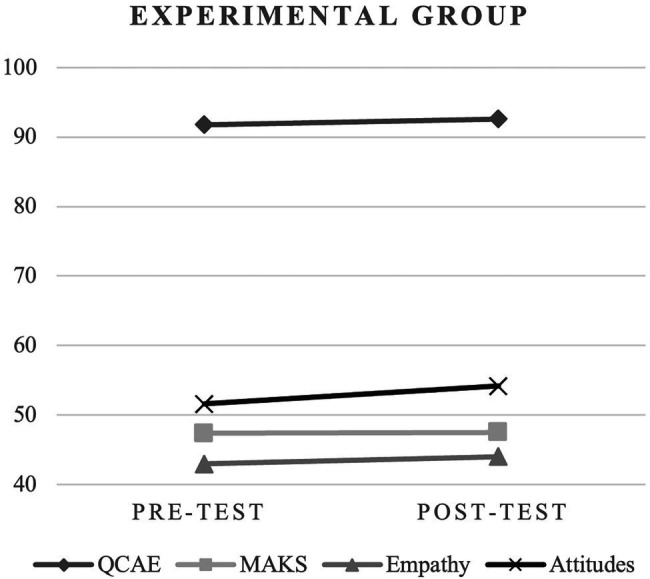
Comparative analysis in experimental group.

Regarding the Total Score of the QCAE (Table 2; Figures 2, 3), in both groups, there was no statistical significance between moments, although there were differences between the group difference (3.39 ± 1.89), *t*_(100)_ = 1,795, *p* = 0.038, Cohen’s *D* = 0.355 (−0.037–0.746). However, regarding cognitive empathy, there was a statistical difference between pre- and post-test only in the control group.

**Table 2:** Comparative analysis of the means for the pre- and post-test Questionnaire of Cognitive and Affective Empathy (QCAE) in the control and experimental groups (AEC: Affective Empathy-Emotion Contagion; AEPR: Affective Empathy-Proximal responsivity; AEPRR: Affective Empathy-Peripheral Responsivity; CEPT: Cognitive Empathy-Perspective Taking; and CEOS: Cognitive Empathy-Online Simulation).

Dimensions			Intrasubject				Interaction			
			Pre-test (M1)	Post-test (M2)	*p*	Cohen’s *D*	Dif_M2_M1	Mean difference	*p*	Cohen’s *D*
Affective Empathy	AEC	Control Group	12.65 ± 2.24	12.82 ± 2.56	0.295	-	0.18 ± 0.33	0.12 ± 0.41	0.387	-
		Experimental Group	12.49 ± 1.92	12.78 ± 2.25	0.116	-	0.29 ± 0.24			
	AEPR	Control Group	12.29 ± 2.48	12.02 ± 2.46	0.186	-	−0.27 ± 0.30	0.14 ± 0.37	0.356	-
		Experimental Group	12.63 ± 1.97	12.49 ± 2.41	0.260	-	−0.14 ± 0.21			
	AEPRR	Control Group	11.65 ± 2.46	11.31 ± 2.44	0.163		−0.33 ± 0.34	0.25 ± 0.42	0.275	-
		Experimental Group	11.37 ± 2.62	11.29 ± 2.93	0.382	-	−0.08 ± 0.26			
	TOTAL	Control Group	36.59 ± 6.00	36.16 ± 6.04	0.246	-	−0.43 ± 0.62	0.51 ± 0.81	0.264	-
		Experimental Group	36.49 ± 4.56	36.57 ± 6.27	0.439	-	0.08 ± 0.51			
Cognitive Empathy	CEPT	Control Group	28.67 ± 6.05	27.25 ± 7.19	0.033	-0.263	−1.41 ± 0.75	1.65 ± 0.90	0.036	0.361
		Experimental Group	30.02 ± 4.87	30.25 ± 5.33	0.321	-	0.24 ± 0.50			
	CEOS	Control Group	27.10 ± 5.26	25.98 ± 5.45	0.025	−0.282	−1.12 ± 0.56	1.24 ± 0.74	0.049	0.331
		Experimental Group	28.31 ± 3.97	28.43 ± 4.78	0.405	-	0.12 ± 0.49			
	TOTAL	Control Group	55.76 ± 10.42	53.24 ± 11.96	0.017	-0.306	−2.53 ± 1.16	2.88 ± 1.46	0.026	0.391
		Experimental Group	58.33 ± 7.41	58.69 ± 8.96	0.347	-	0.35 ± 0.89			
TOTAL		Control Group	88.71 ± 12.13	87.39 ± 14.33	0.185	-	−2.96 ± 1.51	3.39 ± 1.89	0.038	0.355
		Experimental Group	91.80 ± 8.69	92.63 ± 10.43	0.228	-	0.43 ± 1.14			

Concerning Total Score of the MAKS (Table 3; Figures 2, 3), in both groups, there was no statistical significance between moments. Differences were found between the two moments (pre- and post-test) in the experimental group.

**Table 3:** Comparative analysis of the means for the pre- and post-test Mental Health Knowledge Schedule (MAKS) in the control and experimental groups.

		Intrasubject				Interaction			
		Pre-test (M1)	Post-test (M2)	*p*	Cohen’s *D*	Dif_M2_M1	Mean difference	*p*	Cohen’s *D*
MAKS I	Control Group	21.98 ± 2,88	22.39 ± 3.24	0.139	-	0.41 ± 0.38	0.27 ± 0.50	0.489	-
	Experimental Group	23.39 ± 2.93	24.08 ± 2.88	0.022	0.291	0.69 ± 0.33			
MAKS II	Control Group	24.25 ± 3.84	23.98 ± 1.99	0.239	-	−0.27 ± 0.38	−0.29 ± 0.48	0.292	-
	Experimental Group	23.98 ± 3.03	23.41 ± 1.82	0.027	−0.276	−0.57 ± 0.29			
MAKS TOTAL	Control Group	46.24 ± 4.91	46.37 ± 402	0.396	-	0.14 ± 0.52	−0.02 ± 0.71	0.271	-
	Experimental Group	47.37 ± 4.51	4749 ± 3.49	0.404	-	0.11 ± 0.48			

Regarding Empathy variable (Table 4; Figures 2, 3), in the control group, the scores from the pre-test were 44.14 ± 11.27 and the post-test were 46.61 ± 10.54, there was statistical significance between moments, *t*_(50)_ = 1.99, *p* = 0.026, Cohen’s *D* = 0.279 (−0.002–0.558). In the experimental group, although there was an increase in the mean (pre-test: 42.98 ± 9.97; post-test: 44.00 ± 10.67), it was not statistically significant.

**Table 4:** Comparative analysis of the means for the pre- and post-test variables Empathy and Attitudes in the control and experimental groups.

		Intrasubject				Interaction			
		Pre-test (M1)	Post-test (M2)	*p*	Cohen’s *D*	Dif_M2_M1	Mean difference	*p*	Cohen’s *D*
Empathy	Control Group	44.14 ± 11.27	46.61 ± 10.54	0.026	0.279	2.47 ± 1.24	−1.45 ± 1.74	0.203	-
	Experimental Group	42.98 ± 9.97	44.00 ± 10.67	0.203	-	1.02 ± 1.22			
Attitudes	Control Group	51.55 ± 6.70	52.94 ± 6.23	0.021	0.294	1.39 ± 0.66	1.18 ± 1.00	0.122	-
	Experimental Group	51.61 ± 6.96	54.18 ± 6.24	<0.001	0.478	2.57 ± 0.75			

In the Attitudes variable (Table 4; Figures 2, 3), in the experimental group, the scores from the pre-test were 51.61 ± 6.96 and the post-test were 54.18 ± 6.24, there is statistical significance between moments, *t*_(50)_ = 3,415, *p* = <0.001, Cohen’s *D* = 0,478; (0.186–0.766). In the control group, the scores from the pre-test were 51.55 ± 6.70 and the post-test were 52.94 ± 6.23, there was statistical significance between moments, *t*_(50)_ = 2.09, *p* = 0.021, Cohen’s *D* = 0.294 (0.012–0.573). The values for the experimental group were higher.

Concerning heart rate, it was found that there are no statistically significant differences between the control group and the experimental group regarding the mean, maximum, and minimum. However, as for electrodermal activity (EDA), the differences observed between the two groups were statistically significant (Table 5). It was also found a higher number of SCR responses in the control group compared to the experimental one, which was unexpected.

**Table 5:** Summary of the statistical measures related to Heart Rates and electrodermal activity in the control and experimental groups.

		Mean/*SD*	*p*-value
Heart rate Average	Control Group	94.50 ± 20.12	0.286
	Experimental Group	116.35 ± 143.89	
Heart rate Minimum	Control Group	72.26 ± 16.01	0.778
	Experimental Group	71.40 ± 14.71	
Heart rate Maximum	Control Group	120.80 ± 20.91	0.482
	Experimental Group	123.69 ± 20.48	
Number of skin conductance responses	Control Group	66.73 ± 17.49	0.011^*^
	Experimental Group	55.65 ± 24.85	

Data are presented as mean (SD).

* Differences in *p*-value after application of *T*-test.

## Discussion

Virtual Reality is an instrument that overcomes several limitations of conventional methods to try to improve empathy using primarily the perspective-taking method. Consistent with other similar studies, the results show that the use of virtual reality led to an increase in positive attitudes (Kalyanaraman et al., 2010; Schutte and Stilinović, 2017; Bertrand et al., 2018; Herrera et al., 2018).

The fact that participants in the experimental group actively interacted and embodied the virtual representation of the person diagnosed with schizophrenia and the control did not have the same presence may have been an important factor for the participants’ social identity and consequently contributed to a change in the attitudes processes that leads to a variation in the empathic mechanism (Herrera et al., 2018; Schoeller et al., 2019; Rueda and Lara, 2020). A study by Lara and Rueda (2021) states that virtual embodiment by its characteristics may lead to more significant concern for the specific target that translates into effective changes in behavior and, therefore, a change in attitudes.

The indication of the results after the exposure of the experimental group in the Affective and Cognitive Empathy was expected for the experimental group, considering that the empathic dimensions (cognitive and affective) are related (Rueda and Lara, 2020). Similarly, both groups increased empathic feelings toward people diagnosed with schizophrenia after the experience, even though in the experimental group this improvement was not statistically significant. These results are partially supported by the existing literature, which indicates that after performing a perspective-taking task, participants tend to feel more empathy toward a specific target (Bearman et al., 2015; Herrera et al., 2018; Loon et al., 2018; Martingano et al., 2021). In this study, many participants had limited or no existing exposure to VR, and they could have been more focused on the virtual reality experience itself than on the stimuli presented. Also, 2D videos are more familiar to participants and the accommodation to the technological apparatus of VR could have an impact on the results obtained as well.

The results also indicate a significant positive variation in the variable Attitudes between moments, more evident in the experimental group, as expected. The change in empathy in both groups may be directly associated with positive attitudes toward people diagnosed with schizophrenia. These results are corroborated by the literature, which indicates that an increase in empathic feelings related to a specific target result in changes in attitude toward that target (Batson et al., 1997; Batson, 2009; Herrera et al., 2018; Bujić et al., 2020; Formosa et al., 2020).

It is important to consider that promoting more empathetic attitudes is complex and there is a need to understand what influences and facilitates that change. One of the factors that can vary the level of empathy is the time before the change. No significant differences were found regarding QCAE in the experimental group, and the reason could be the short exposure time (Tong et al., 2020). Furthermore, a 2021 metaanalysis found that VR increases the emotional empathy of its users, but not their cognitive empathy (Martingano et al., 2021). In our study, differences in cognitive empathy occurred only in the control group.

Even though the experimental group did not have any specific information in relation to diagnostic symptomology of schizophrenia, participant’s performance on MAKS was significantly higher post-intervention, which corroborates another study carried out in the field of psychology training (Formosa et al., 2020).

Regarding psychophysiological parameters, for HR, there were no statistically significant differences. Despite this, both groups showed high values (60–100 bpm), which could indicate that the participants got stressed with the task (Todd et al., 2015). According to Andreassi (2006), the acceleration and deceleration of heart rate may present itself as a competitive and defensive response. This mechanism may underlie the variability between the mean maximum and minimum HR values that were verified. Thus, according to this analysis, the results suggest that participants in both groups triggered responses to the experience’s stimuli, which may have influenced the increase in empathy (Andreassi, 2006).

The EDA is an autonomic response influenced by the sympathetic nervous system resulting from environmental stimuli. It increases with emotional responses such as excitement or nervousness (Andreassi, 2006). It can be analyzed through the skin conductance response (non-specific SCR), which corresponds to rapid transient events in the ADS signal and can be expressed as the number of responses per minute (Andreassi, 2006; Braithwaite et al., 2013). Typical values of non-specific SCRs are 1–3/min (Braithwaite et al., 2013). In the present study, the number of SCR was significantly different between the groups, it should be noted that both had responses/min above normal values. One of the reasons why this happened could be related to the time of exposure to the 2D stimulus and the ambient temperature of the space since the experiments were carried out on different days and the temperature may have induced different results. Moreover, it is important to note that uncontrollable factors during the measurement of these signals, such as ambient temperature or the participant’s state, may have influenced the values of EDA and HR (Jang et al., 2015; Zangróniz et al., 2017). Additionally, although the content of these experiences is curated, participants have some degree of choice as to where to look in the experimental group, whereas in 2D video, the participants looked straight ahead. Since virtual reality can present some barriers (usability challenges, cybersickness, or costs), 2D videos may also be a possible solution to increase empathy toward people with schizophrenia, according to our results, if these videos are realistic.

Our study has some limitations. One of the limitations of this study is the absence of a group without any exposure to compare the efficiency of the use of technologies to promote empathy. The authors propose future studies that use technology as a method of promoting empathy through mental health problems, using a condition that has no technological exposure. Another limitation was the sample size of women and men that were imbalanced since some indications suggest that there are differences between women and men regarding empathy because women are positively associated with emotional empathy, whereas males are negatively associated with cognitive empathy (Christov-Moore et al., 2014). A further limitation in this study is that the immersive nature of virtual reality has not been assessed; as a result the research team is not aware of the impact of this aspect on the change in empathy and the other variables. A usability test should have been conducted to determine the ease of use, usefulness, and perceived quality and sense of realism of the VR experiment.

It is suggested that research in this area continues to collect more solid evidence with more significant samples, more control over the variables under study, and follow-up to understand whether empathy levels and positive attitudes are maintained over time. It is essential not only to identify the best way to define and measure empathy but also to understand how to design VR experiences to enhance empathy.

## Conclusion

Both interventions achieved higher levels of empathy and improved attitudes toward people diagnosed with schizophrenia. However, in this study, virtual reality appears to be most effective in inducing the participant’s reaction.

Empathy is vital for positive human interaction and has been shown in previous research to be associated with increased positive attitudes and, therefore, prosocial behavior.

Virtual reality provided a more immersive and interactive experience, leading participants to adopt a person with schizophrenia’ perspective. In addition, this study has fostered an increase in positive attitudes and more empathetic responses toward people diagnosed with schizophrenia. Therefore, it is understood that not only virtual reality experiences, but also realistic 2D videos, have the potential to reduce some prejudices and discriminatory attitudes and can be an effective tool to educate and raise awareness of schizophrenia among university students, especially those that will work in the health field. Nevertheless, it is necessary to better understand inherent processes. Considering the lack of evidence in this area, more robust research on the guidelines, effectiveness, and acceptability of virtual reality programs for schizophrenic empathy should be conducted.

## Data Availability Statement

The raw data supporting the conclusions of this article will be made available by the authors, without undue reservation.

## Ethics Statement

The studies involving human participants were reviewed and approved by Ethics Committee of the School of Health—Polytechnic of Porto. The patients/participants provided their written informed consent to participate in this study.

## Author Contributions

All authors contributed to the conception and design of the study. MA, AM, and PG collected the data, organized the database, and performed the statistical analysis. All authors contributed to the article and approved the submitted version.

## Funding

This work was supported by Fundação para a Ciência e Tecnologia (FCT) through R&D Units funding (UIDB/05210/2020).

## Conflict of Interest

The authors declare that the research was conducted in the absence of any commercial or financial relationships that could be construed as a potential conflict of interest.

## Publisher’s Note

All claims expressed in this article are solely those of the authors and do not necessarily represent those of their affiliated organizations, or those of the publisher, the editors and the reviewers. Any product that may be evaluated in this article, or claim that may be made by its manufacturer, is not guaranteed or endorsed by the publisher.
